# ANP32B Deficiency Protects Mice From Lethal Influenza A Virus Challenge by Dampening the Host Immune Response

**DOI:** 10.3389/fimmu.2020.00450

**Published:** 2020-03-13

**Authors:** Sebastian Beck, Martin Zickler, Vinícius Pinho dos Reis, Thomas Günther, Adam Grundhoff, Patrick T. Reilly, Tak W. Mak, Stephanie Stanelle-Bertram, Gülşah Gabriel

**Affiliations:** ^1^Viral Zoonosis - One Health, Heinrich Pette Institute, Leibniz Institute for Experimental Virology, Hamburg, Germany; ^2^Institut Clinique de la Souris, University of Strasbourg, Illkirch-Graffenstaden, France; ^3^University Health Network, Toronto, ON, Canada; ^4^Institute for Virology, University of Veterinary Medicine, Hanover, Germany

**Keywords:** influenza A virus, pathogenesis, ANP32A, ANP32B, antiviral immunity

## Abstract

Deciphering complex virus-host interactions is crucial for pandemic preparedness. In this study, we assessed the impact of recently postulated cellular factors ANP32A and ANP32B of influenza A virus (IAV) species specificity on viral pathogenesis in a genetically modified mouse model. Infection of ANP32A^−/−^ and ANP32A^+/+^ mice with a seasonal H3N2 IAV or a highly pathogenic H5N1 human isolate did not result in any significant differences in virus tropism, innate immune response or disease outcome. However, infection of ANP32B^−/−^ mice with H3N2 or H5N1 IAV revealed significantly reduced virus loads, inflammatory cytokine response and reduced pathogenicity compared to ANP32B^+/+^ mice. Genome-wide transcriptome analyses in ANP32B^+/+^ and ANP32B^−/−^ mice further uncovered novel immune-regulatory pathways that correlate with reduced pathogenicity in the absence of ANP32B. These data show that ANP32B but not ANP32A promotes IAV pathogenesis in mice. Moreover, ANP32B might possess a yet unknown immune-modulatory function during IAV infection. Targeting ANP32B or its regulated pathways might therefore pose a new strategy to combat severe influenza.

## Introduction

Influenza A virus (IAV) cross-species transmission from animal-to-man poses a continuous risk to global health. The next influenza pandemic may cause an estimated 80 million deaths worldwide according to a recent report by the World Health Organization (www.who.int). Thus, understanding the molecular basis of interspecies transmission is crucial for pandemic preparedness and for the development of efficient countermeasures and treatments.

Interspecies transmission of IAV requires a complex interplay of host adaptive mutations in the viral genome and their dynamic interactions with host cell factors. A major determinant herein is the heterotrimeric viral polymerase consisting of three subunits: PB1, PB2, and PA (3P). The 3P polymerase together with the viral nucleoprotein (NP) and the viral RNA form the viral genome as viral ribonucleoprotein (vRNP) complexes.

Host adaptive mutations in the polymerase subunit PB2 (such as E627K and D701N) may confer high polymerase activity and replicative fitness in mammalian cells as well as elevated mammalian pathogenicity and efficient mammal-to-mammal transmission (1). Molecular, biochemical and crystallographic evidence revealed that both host adaptive PB2 mutations pose, at least in part, adaptations to the importin-α nuclear import machinery of the mammalian cell (2–4). Herein, importin-α1 and -α7 facilitate the nuclear transport of monomeric PB2 701N or vRNPs, thereby mediating increased IAV replication in mammalian cells (2, 5). Importin-α7 additionally promotes virus replication by a yet unknown mechanism beyond nuclear transport, supporting high-level IAV replication in various mammalian, including human cells (3). Most importantly, it could be shown that mice lacking the importin-α7 gene can no longer support high-titer virus replication of PB2 627K or 701N adapted influenza strains in the murine respiratory tract, leading to up to 100% survival upon influenza challenge (3, 6–8).

More recently, another host factor was proposed to play a crucial role in IAV PB2 627K mediated interspecies transmission. The ANP32 (acidic leucine-rich nuclear phosphoprotein 32 kDa) protein family, which mainly consists of ANP32A, ANP32B, and ANP32E, regulates many different cellular processes, including chromatin architecture, mRNA export, and apoptosis (9). In 2016, ANP32A (alias pp32) and ANP32B (alias APRIL) were identified as positive-regulatory factors of IAV polymerase activity and replication in human cell lines (10). Both, ANP32A and ANP32B proteins were originally described as co-factors for IAV cRNA-to-vRNA viral replication *in vitro*, albeit the underlying molecular mechanisms remain still elusive (11). It was further demonstrated that an avian-type polymerase (PB2 627E), which is naturally restricted in mammalian cells, could be rescued by co-expressing the avian ANP32A homologue (10, 12). The deficiency of human ANP32A to promote avian-type polymerase activity in human cells was pinpointed to a 33 amino acid insertion in the avian homologue (10). Furthermore, there is accumulating evidence that species-specific differences in the ANP32 proteins might affect their ability to promote viral replication in human cell culture systems (13, 14). However, it is currently unknown whether ANP32A and ANP32B may also affect viral pathogenicity in a mammalian animal model and are thus of biological relevance.

In this study, we aimed to elucidate the impact of ANP32A and ANP32B on influenza A virus pathogenicity. Therefore, we infected mice lacking either the ANP32A or ANP32B gene with H3N2 or H5N1 IAV containing the PB2 627K host-adaptive signature. We combined animal data with genome-wide transcriptome analyses to elucidate the role of ANP32A and ANP32B on influenza virus infection and immunity.

## Materials and Methods

### Biosafety and Ethics Statement

Influenza A virus infections of cell lines or mice were carried out in BSL-2 (H3N2) or BSL-3 (H5N1) facilities at the Heinrich Pette Institute following standard operation procedures.

All animal experiments conducted in this study were in strict accordance with the guidelines of the German animal protection law. All protocols used were approved by the German authorities (Behörde für Stadtentwicklung und Umwelt Hamburg, licensing number: 08/17).

### Cell Lines and Cell Culture

HeLa cells (ATCC, CCL-2, female) and MDCK (Madine-Darby canine kidney) cells (ATCC, CCL-34, female) were either grown in Dulbeco's Modified Eagles Medium (DMEM) or Minimal Essential Medium (MEM), respectively, both supplemented with 10% fetal bovine serum (FBS), 1% L-glutamine (L-Glu) and 1% penicillin/streptomycin (P/S). ANP32B knockout and control HeLa cell lines were generated using the CRISPR-Cas technique and kindly provided by Jan Chemnitz/Joachim Hauber, Heinrich Pette Institute, Hamburg, Germany. Lung fibroblasts (LFs) were isolated from ANP32A^−/−^ and ANP32B^−/−^ male mice as well as their wild type male litter mates and spontaneously immortalized by serial passaging. The isolation procedure is described in more detail below. Murine LFs were cultivated in DMEM medium, supplemented with 10% FBS, 1% L-Glu, 1% P/S, 1% sodium pyruvate, and 1% non-essential amino acids. All cell lines were maintained in a temperature-controlled incubator at 37°C, 5% CO_2_ and 95% relative humidity (rH). Human HeLa cell lines were verified by STR analysis (Eurofins Genomics) and all cell lines were regularly checked to be mycoplasma negative (*Venor*®*GeM Classic Mycoplasma PCR Detection Kit*; Minerva Biolabs GmbH).

### Viruses

Influenza A virus strains used in this study include A/Aichi/2/68 (H3N2), A/WSN/33 (H1N1) and A/Vietnam/1194/04 (H5N1). The A/Aichi/2/68 virus strain is a reassorted virus carrying HA and NA from the 1968 pandemic Aichi lineage and the remaining six gene segments from A/WSN/33 (hereafter referred to as H3N2). This mouse-adapted H3N2 virus has been shown to be pathogenic in mice, in contrast to currently circulating H3N2 variants (15, 16). The A/Vietnam/1194/04 (H5N1) virus is a human isolate from a fatal human case in Vietnam in 2004. This virus was kindly provided by Hans-Dieter Klenk, University of Marburg, Marburg, Germany. All virus strains used in this study carry the human-adaptive PB2 627K signature, which was verified by Sanger Sequencing. Virus stocks were propagated and grown on MDCK cells. Virus titers were determined by plaque test on MDCK cells following established protocols.

### Animal Models

Mice used in this study were bred and housed under specific pathogen-free (SPF) conditions at the Heinrich Pette Institute, Leibniz Institute for Experimental Virology, Hamburg, Germany. All mice used for infection experiments were housed in individually ventilated cages (IVC). If possible, equal numbers of 10- to 12-week-old female and male mice were used for all infection experiments.

To generate the C57BL/6J ANP32A^−/−^ mouse line and the wild type litter mates (ANP32A^+/+^) thereof, sperm was purchased from the European Mouse Mutant Archive (EMMA) depository (*EM:07238*) (17) and used for embryo transfer at the university medical center Hamburg-Eppendorf, Hamburg, Germany.

C57BL/6J ANP32B^−/−^ mice and the wild type litter mates (ANP32B^+/+^) thereof were generated as recently described using Cre recombinase fused to the ligand binding domain (LBD) of estrogen receptor (Cre-ER_LBD_) (18). Since germline knockout of ANP32B is lethal, a conditional knockout approach was chosen. Herein, the knockout was induced by feeding Tamoxifen-containing nutrients (LASCRdiet CreActive TAM400 (400 mg/kg); Genobios) to 6-week old mice for ~4–weeks. All litter mates of the ANP32B mouse strain were fed tamoxifen-containing food pellets, but knockout of ANP32B was only induced in those litter mates expressing the Cre-ER_LBD_ construct. Genotyping of all mice was performed using PCR on genomic DNA extracted from ear stamps. The knockout efficiency in the organs of interest (lung, brain) was further verified on protein level by Western blotting.

### Animal Infection Experiments

For infection experiments, mice were briefly placed in isoflurane narcosis (Abbott) following intraperitoneal narcosis with a mixture of ketamine (100 mg/ml, 20 μl per mouse; WDT) and xylazine (20 mg/ml, 10 μl per mouse; WDT), prepared in 0.9% of sterile sodium chloride solution (B. Braun Melsungen GmbH). The narcosis volume was always matched to the weight of each individual animal. The virus inoculum was prepared by appropriate dilution of virus stock in PBS, and mice were infected intranasally with 50 μl of virus inoculum or PBS control inoculum.

For weight loss and survival experiments, mice were monitored for 14 days and the weight of each individual animal was daily recorded. Mice were euthanized at the humane endpoint of 25% loss of original weight at infection day 0.

For organ harvesting experiments, at 3 or 6 days post infection, PBS or virus infected mice were briefly placed in isoflurane narcosis following collection of blood by retrobulbary bleeding and euthanization by cervical dislocation. For total RNA isolation, lungs were removed, preserved in RNAlater RNA stabilization reagent (Qiagen) for 24 h, and stored at −80°C until further processing. For histological examination, a single lung lobe was stored in 4% paraformaldehyde (PFA) solution (in PBS) at 4°C. For determination of virus titers and measurement of cytokines, lung and brain tissue was removed and the weight was recorded. Then, tissue was directly homogenized in PBS, aliquoted and stored at −80°C. Viral titers were determined by plaque test on MDCK cells as described below.

### Isolation and Cell Culture of Murine Lung Fibroblasts

Male ANP32A^−/−^ or ANP32B^−/−^ mice and the wild type male litter mates thereof were briefly placed in isoflurane narcosis, final retrobulbary bleeding was performed and mice were euthanized by cervical dislocation. Lungs from two mice of each genotype were removed, washed once with PBS, cut into small parts and mixed together. The tissue was digested with collagenase D (in DMEM medium; Sigma-Aldrich/Merck) for 1 h at 37°C, followed by digestion with DNase I (in DMEM medium; Sigma-Aldrich/Merck) for 10 min at room temperature (RT). Then, the tissue was squeezed through a 45 μM mesh filter using DMEM medium, and the cell suspension was centrifuged at 300 × g for 10 min at 4°C. The pelleted cells were resuspended in LF growth medium and seeded into 24-well plates. Cells were incubated for 72 h at 37°C. After 72 h, cells were trypsinized for a few minutes to separate the fibroblasts from the epithelial cells, which are more resistant to trypsin. Cells were then passaged twice a week by differential trypsination until only fibroblast cells remained. These fibroblast cells were further maintained in culture until spontaneous immortalization occurred after ~25 passages, as evident by fast and contact-independent growth of the cells. Knockout of the respective ANP32 protein was verified by Western blotting every five to six passages.

### Influenza A Virus Infection

In order to study viral replication kinetics in human or murine cells (HeLa and LFs, respectively) lacking either ANP32A or ANP32B, cells were seeded in 6-well plates for 16 h and infected using standard protocols. Briefly, virus inoculum was prepared by diluting the virus stock to the desired multiplicity of infection (MOI) in the respective inoculation medium for each cell line (growth medium, without FBS). HeLa cells were infected with both H3N2 and H5N1 at a MOI of 0.1, while murine LFs were infected at a MOI of 1.0 with both virus strains. The medium was removed from the cells, cells were washed once with PBS and 500 μl of virus inoculum was added. Cells were incubated for 30 min at 37°C to allow adherence of virus particles. Then, inoculum was removed, cells were washed three times with PBS and the appropriate infection medium (inoculation medium, supplemented with 0.2% BSA, 0.1% FBS, and 1:1,000 diluted TPCK-treated trypsin (Sigma-Aldrich/Merck), if necessary) was added. Cell culture supernatants were collected over a time period of 72 h and stored at −80°C. Viral titers were determined by plaque test on MDCK cells as described below.

Viral nucleoprotein (NP) expression was analyzed in ANP32B deficient HeLa cells 24 h post infection. Therefore, HeLa cells (Ctrl, ANP32B KO #1, and ANP32B KO #2), seeded in 6-well plates the day before, were infected with H3N2 IAV at a MOI of 1.0. The detailed infection protocol is described above. After 24 h, the medium was removed from the cells, cells were washed once with PBS, cell lysates were prepared and analyzed for nucleoprotein expression by Western blotting. Quantification of NP expression was performed in two independent experiments using ImageJ software.

### Plaque Test

A plaque test on MDCK cells was performed to determine viral titers of virus stocks, in cell culture supernatants or in organ homogenates. Briefly, MDCK cells were seeded in 24- or 6-well plates for 24 h. Virus containing supernatants/homogenates were serially diluted 10-fold in PBS. Medium was removed from seeded cells, cells were washed once with PBS, and virus dilutions were directly added to the cells. After 30 min incubation at 37°C, overlay medium (2xMEM, supplemented with 2% L-Glu and 2% P/S, mixed 1:1 with 2.5% Avicel solution) was added to the cells without removing the virus inoculum, and cells were incubated for 72 h at 37°C. Then, overlay medium was removed, cells were fixed with 4% PFA solution at 4°C for at least 30 min, and counterstaining was performed with crystal violet solution. Plaques were counted and virus titers, presented as plaque forming units (PFU) per ml or per gram of organ weight, were further determined manually.

### Immunohistochemistry

Histopathological changes were analyzed in the lungs of H3N2 and H5N1 IAV infected mice at 3 and 6 days post infection. PBS-treated mice served as control (*n* = *5* animals per group). Lungs were harvested as described and stored in 4% PFA solution at 4°C until further processing. Then, lungs were thin-sectioned, deparaffinized and either stained with hematoxylin/eosin as described before (19) or immunohistochemically (IHC) using a rabbit anti-influenza nucleoprotein antibody (1:10,000; Life Technologies, #PA5-32242). As a secondary antibody for IHC, a biotin-conjugated anti-rabbit IgG antibody (1:200; Jackson Immuno Research; #711-066-152) in combination with the *ZytoChem Plus (HRP) Broad Spectrum (DAB) Kit* (Zytomed systems) was used. Image acquisition was performed on a *Nikon Eclipse 80i upright light microscope* (Nikon, Japan) coupled with *Color Camera Nikon DS-Ri2* (Nikon, Japan) and the *NIS-Elements Advanced Research software* (Nikon; Japan). Images were captured at 10x magnification, three independent fields were taken from each slide and representative images were chosen. Images were further processed using *Adobe Photoshop software* (Adobe Systems Inc.).

### Multiplex Immunoassay

Lung tissue extracted from PBS or virus infected mice was homogenized in PBS and stored at −80°C. A custom-designed multiplex immunoassay to detect murine TNF-α, MCP-1 (CCL-2), IFN-α, IL-1β, IL-6, IL-10, IL-17A, and IL-21 was purchased from Life Technologies GmbH (Assay-ID: *MXNKRYE*). Measurement of cytokine and chemokine levels in lung homogenates was carried out according to manufacturer's instructions. For detection, the *Luminex 200 system* (Bio-Rad) was used.

### Preparation of Lysates From Cell Lines or Murine Tissue

In order to prepare cell lysates for Western blot analysis, ~2 × 10^6^ cells were pelleted by centrifugation (5 min, 2,000 × g, 4°C) and lysed for 20 min (4°C) using an appropriate amount of HEPES lysis buffer (50 mM HEPES pH 8.0, 200 mM NaCl, 0.5% Igepal, 25% glycerol, 0.07 μl/ml β-mercaptoethanol), supplemented with a protease/phosphatase inhibitor cocktail (HALT; Life Technologies GmbH). Cell debris were removed by centrifugation (10 min, 16,000 × g, 4°C), the supernatant was mixed with 4 x Laemmli Loading Dye, heated to 95°C for 5 min and stored at −20°C.

Cell lysates derived from murine organ tissue (lung, brain) were prepared by homogenization of ~30 mg of extracted tissue in HEPES lysis buffer using a RETCH homogenizer (20 Hz, 2 min; 30 Hz, 1 min). To allow complete cell lysis, the suspension was incubated on ice for 20 min, following centrifugation (20 min, 20,000 × g, 4°C) to remove cell debris. Supernatants were mixed with 4 x Laemmli Loading Dye, heated to 95°C for 5 min and stored at −20°C.

### Western Blotting

Western blotting was performed to detect ANP32 proteins or viral nucleoprotein in cell lysates or organ homogenates. Briefly, lysates were separated on 10% SDS polyacrylamide gels following transfer on nitrocellulose membranes. The membranes were blocked with 3% BSA in PBS-T (phosphate buffered saline, supplemented with 0.1% Tween-20) for 1 h at RT. Primary antibody incubation (dilution 1:1,000, in Superblock reagent; Life Technologies GmbH) was carried out over night at 4°C. Primary antibodies used in this study include: anti-GAPDH (Cell Signaling, #2118); anti-phospho-ANP32B (20), anti-ANP32B (Santa Cruz, #sc-68219), anti-influenza-nucleoprotein (Abcam, #ab128193); anti-ANP32A (Santa Cruz, #sc-5652). Membranes were incubated with appropriate secondary antibodies (dilution 1:20,000, in PBS-T) for 1 h at RT. Secondary antibodies include (all from Sigma-Aldrich/Merck): anti-mouse IgG-HRP (#A4416), anti-rabbit IgG-HRP (#A8275) and anti-goat IgG-HRP (#A5420). Imaging was performed using the *SuperSignal West Femto Maximum Sensitivity Substrate* (Life Technologies GmbH) and the bioimaging system LAS4000 (GE Healthcare). Images were further processed using *ImageJ* and/or *Adobe Photoshop software*.

### RNA Isolation

Lung tissue isolated from PBS or virus infected mice (*n* = *3* animals per group) was stored in RNA later reagent (Qiagen) for 24 h. Equal parts (~30 mg) were distributed into screw-cap tubes filled with metal beads and stored at −80°C until further processing. Total RNA was subsequently extracted using the *innuPrep RNA Mini Kit* (Analytik Jena) according to manufacturer's instructions. An additional on-column treatment with *DNase I* (RNase-free DNase set; Qiagen) was performed to digest genomic DNA. *Ribolock RNAse inhibitor* (Life Technologies) was added to the isolated RNAs and RNAs were stored at −80°C.

### Next Generation Sequencing

Total RNA derived from three animals per group/condition was used for next generation sequencing (NGS). RNA quality was assessed using *Agilent RNA 6000 Nano Kit* (Agilent Technologies) and high-quality RNA (RIN ≥ 7) was used for NGS analysis. Polyadenylated mRNA was selectively purified using *NEBNext Poly(A) mRNA Magnetic Isolation Module* (New England Biolabs). Illumina compatible sequencing libraries were prepared using *NEXTflex rapid Directional qRNA-Seq Kit (*Bioo Scientific) and quality of libraries was assessed using *Agilent DNA 1000 Kit* (Agilent Technologies). Sequencing was performed on a NextSeq500 (Illumina) with *NextSeq 500/550 High Output Kit v2.5* (75 cycles SE, Illumina).

High quality FASTQ data was aligned to the annotated human reference genome hg38 (https://www.ncbi.nlm.nih.gov/assembly/GCA_000001405.28) using STAR (21). Quantification of gene counts was performed with the build in gene quantification function of STAR. Differentially expressed genes (DGEs) were identified by DeSeq2 (22) using significance and log2FoldChange (LFC) cutoffs (FDR < 0.1; LFC ≥ 1 or ≤ −1). Significantly disregulated genes were evaluated manually according to their associated gene ontology (GO) terms. Genes presented in the top10 of significantly altered GOs were further visualized using heatmaps, created with the online tool *ClustVis* (23). LFC cutoffs were applied as indicated in the figure legend.

### Quantitative Real-Time Reverse Transcription PCR (qRT-PCR)

Total RNA isolated from lung tissue of PBS or H3N2 IAV infected ANP32B^+/+^ and ANP32B^−/−^ mice was reverse transcribed into cDNA using a *Oligo p(dN)9 Random nonamer* primer mix (Gene Link) and *SuperScript III Reverse Transcriptase* (SS-RT; Life Technologies GmbH) according to manufacturer's instructions. Briefly, 500 ng of RNA was mixed with dNTPs (Life Technologies) and p(dN)9 primer mix and incubated for 5 min at 65°C. Then, a mix composed of SS-RT, RNase inhibitor (Ribolock; Life Technologies) and DTT (Life Technologies) was added and the cDNA synthesis was carried out in a PCR cycler (input parameters: 25°C/5min, 50°C/60min, 70°C/15min). RNase-free water was added to the cDNA to obtain a sufficient volume for the quantitative qPCR reaction, and cDNAs were stored at −20°C. qRT-PCR was employed to determine the expression levels of viral nucleoprotein (NP) RNA or to measure the expression levels of cytokines/chemokines, antiviral transcription effectors and effector proteins in the lungs of PBS-treated or H3N2 IAV infected mice at 3 dpi. Therefore, the *FastStart Essential DNA Green Master* kit (SYBR green; Roche diagnostics) in combination with the LightCycler® 96 system (Roche diagnostics) was used according to manufacturer's instructions. In brief, a master mix composed of primers (sequences shown below) and SYBR green reagent was distributed into a 96-well plate and cDNAs were added. The cycling parameters were set as follows: 95°C/5 min; 45 cycles of 95°C/15 s, 60°C/15 s and 72°C/20 s; 95°C/60 s. Success and specificity of the cycling reaction were confirmed using primer melting curves and agarose gel electrophoresis of the obtained PCR products. Obtained Ct values were further evaluated manually using murine RPS9 for normalization and the ΔΔCt method (24).

Sequences of primers used for qRT-PCR:

*H1N1_NP_fwd, 5*′*-*AGGGTCAGTTGCTCACAAGTCC-3′
*H1N1_NP_rev*, 5′-TTTGAAGCAGTCTGAAAGGGTCTA-3′
*Murine RPS9_fwd*, 5′-CCGCCTTGTCTCTCTTTGTC-3′
*Murine RPS9_rev*, 5′-CCGGAGTCCATACTCTCCAA-3′
*Murine MCP-1_fwd*, 5′-TGATCCCAATGAGTAGGCTGGAG-3′
*Murine MCP-1-rev*, 5′-ATGTCTGGACCCATTCCTTCTTG-3′
*Murine TNF-*α*_fwd*, 5′-TCGTAGCAAACCACCAAGTG-3′
*Murine TNF-*α*_rev* 5′-AGATAGCAAATCGGCTGACG-3′
*Murine IL-6_fwd*, 5′-CTCCCAACAGACCTGTCTATAC-3′
*Murine IL-6_rev*, 5′-GTGCATCATCGTTGTTCATAC-3′
*Murine IFN-*β*1_fwd*, 5′-GTCTCATTCCACCCAGTGCT-3′
*Murine IFN-*β*1_rev*, 5′-CCAGCTCCAAGAAAGGACGA-3′
*Murine IL-1*β*_fwd*, 5′-GCACTACAGGCTCCGAGATGAAC-3′
*Murine IL-1*β*_rev* 5′-TTGTCGTTGCTTGGTTCTCCTTGT-3′
*Murine CXCL10_fwd*, 5′-ATCATCCCTGCGAGCCTATCCT-3′
*Murine CXCL10_rev*, 5′-GACCTTTTTTGGCTAAACGCTTTC-3′
*Murine IRF-7_fwd*, 5′-CAGCGAGTGCTGTTTGGAGAC-3′
*Murine IRF-7_rev*, 5′- AAGTTCGTACACCTTATGCGG-3′
*Murine Mx-1_fwd*, 5′- GGGGAGGAAATAGAGAAAATGAT-3′
*Murine Mx-1_rev*, 5′- GTTTACAAAGGGCTTGCTTGCT-3′.

### Quantification and Statistical Analysis

All statistical evaluations were performed using the GraphPad Prism 5 v.5.03 software (GraphPad Software, Inc.), and significant statistical differences were further divided based on *p*-values into: ^*^p < 0.05, ^**^p < 0.01, ^***^p < 0.001.

Statistical differences in viral titers between two groups or conditions as well as in quantified Western blot data were determined using the two-tailed Student's *t*-test (*n* ≥ 3 biological replicates). Multiple-comparison statistical analyses of cytokine/chemokine expression data were performed using two-way ANOVA with Bonferroni *post hoc* correction (*n* = 4–7 animals per group).

### Data Availability

All raw data produced in this study are available on request to the corresponding author, Gülsah Gabriel (guelsah.gabriel@leibniz-hpi.de).

The RNA sequencing data generated during this study are deposited at the European Nucleotide Archive (ENA) via the accession number PRJEB35060.

## Results

### ANP32B but Not ANP32A Is Required for Influenza A Virus Pathogenicity in Mice

**Figure 1:**
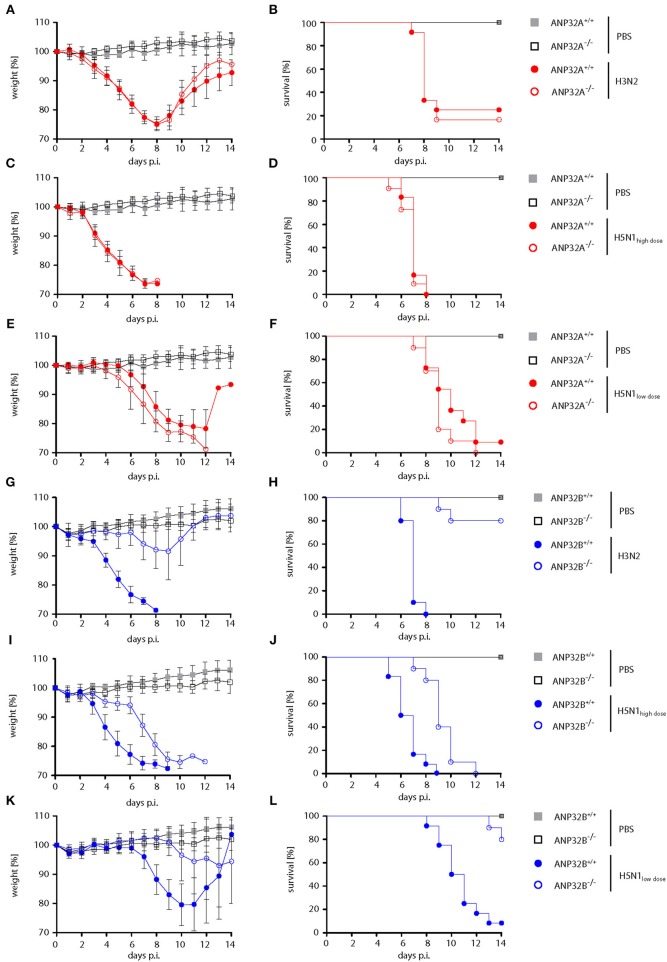
Differential influenza A virus pathogenesis in ANP32A^−/−^ and ANP32B^−/−^ mice. ANP32A^−/−^ (**A–F**; red circles) or ANP32B^−/−^ (**G–L**; blue circles) mice as well as their corresponding wild type litter mates (ANP32A^+/+^ and ANP32B^+/+^, respectively) were either control treated with PBS (gray and black squares) or infected with a seasonal H3N2 subtype (**A,B,G,H**; 10^3^ pfu) or a highly pathogenic H5N1 human isolate (**C,D,I,J**; high dose, 10^3^ pfu; **E,F,K,L**; low dose, 10^1^ pfu). Weight loss **(A,C,E,G,I,K)** and survival **(B,D,F,H,J,L)** were monitored for 14 days post infection (days p.i.). Weight loss data are presented as means ± SD (PBS: *n* = 6–7, virus-infected groups: *n* = 10–12).

First, we wanted to assess whether mammalian ANP32A and ANP32B genes shown to act as positive factors of human-type influenza A virus polymerase activity in cell culture (10, 13, 14) are also involved in viral pathogenesis. Therefore, we infected mice either lacking the ANP32A (ANP32A^−/−^) or ANP32B (ANP32B^−/−^) gene (Figure S1) with human influenza A virus isolates. Infection of ANP32A^−/−^ mice with seasonal H3N2 or human-type H5N1 influenza viruses did not show any differences in weight loss or survival compared to their ANP32A^+/+^ litter mates (Figures 1A–F). In contrast, ANP32B^−/−^ mice infected with H3N2 influenza presented strongly reduced weight loss and lethality compared to their ANP32B^+/+^ litter mates (Figures 1G,H). H3N2 lethality in ANP32B^−/−^ mice was 20% compared to 100% death in ANP32B^+/+^ mice (Figure 1H). ANP32B^−/−^ mice infected with H5N1 at a high dose showed reduced weight loss and delayed death as compared to ANP32B^+/+^ mice (Figures 1I,J). Survival rates in ANP32B^−/−^ mice were significantly increased from 10 to 80% upon low dose H5N1 infection (Figures 1K,L*)*. These findings show that ANP32B but not ANP32A is crucial for H3N2 and H5N1 pathogenicity in mice.

### ANP32B Is Required for Efficient Virus Replication in the Murine Lung

**Figure 2:**
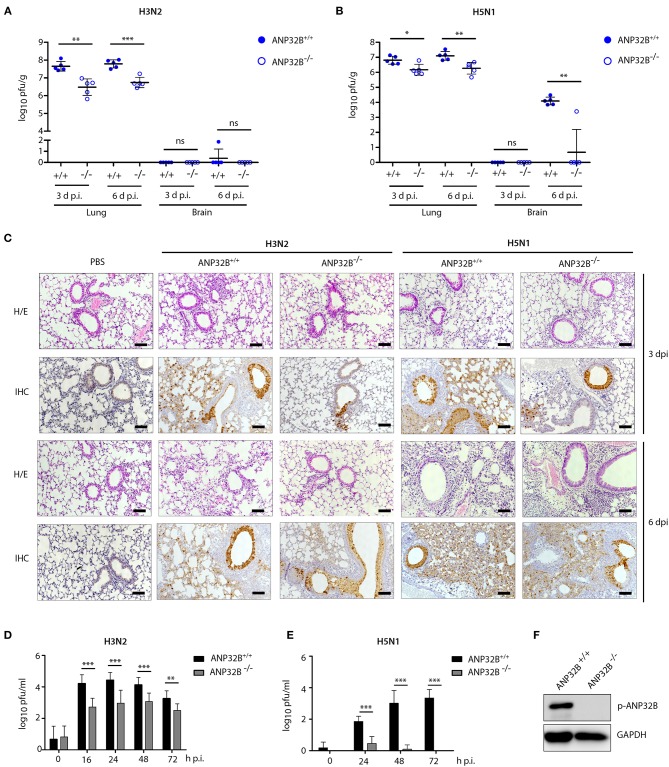
ANP32B deficiency impairs influenza A virus replication in mice and in murine cells. **(A–C)** ANP32B^+/+^ and ANP32B^−/−^ mice were either control treated with PBS or infected with 10^3^ pfu of a seasonal H3N2 subtype **(A)** or a highly pathogenic H5N1 human isolate **(B)**. **(A,B)** Viral titers were determined 3 and 6 days p.i. in lung and brain of infected animals. No virus was detected in PBS infected mice (*n* = 5). Presented are individual organ titers for each animal as well as the means ± SD for each group (*n* = 5–6). **(C)** At 3 and 6 d p.i., lungs from infected animals were removed and immunohistochemically (IHC) stained for viral NP antigen. Additionally, hematoxylin and eosin (H/E) staining was performed. Shown are representative images for each group (*n* = 5). Scale bar represents 10 μm. Original magnification, 10x. **(D,E)** Viral titers in H3N2 or H5N1 infected murine lung fibroblasts deficient for ANP32B were determined by plaque test at the indicated time points (h p.i., hours post infection). **(F)** Knockout of ANP32B in murine lung fibroblasts was confirmed by Western blotting. **(A–F)** Statistical significance was calculated using the two-tailed Student's *t-test* (*p ≤ 0.05, **p ≤ 0.01, ***p ≤ 0.001; ns, not significant).

Next, we analyzed influenza A virus replication in the lungs of mice lacking the ANP32B gene. H3N2 virus replication was reduced 10-fold in the lungs of ANP32B^−/−^ mice compared to ANP32B^+/+^ litter mates (Figure 2A). In line with the respiratory tropism of H3N2, no significant virus replication was detected in the murine brains (Figure 2A). H5N1 virus replication was also reduced 10-fold in the lungs of ANP32B^−/−^ mice compared to ANP32B^+/+^ mice (Figure 2B). H5N1 systemic virus replication was, except for one animal, not detectable in the brains of ANP32B^−/−^ mice on day 6 p.i., compared to ANP32B^+/+^ mice (Figure 2B). As expected, H3N2 and H5N1 virus replication was not altered in mice lacking the ANP32A gene compared to their ANP32A^+/+^ litter mates (Figures S2A–C). This further confirms that ANP32A is not required for viral replication and pathogenicity in mice.

We then continued further characterization of virus tropism and replication in ANP32B^−/−^ and ANP32B^+/+^ mice. There, at 3 and 6 days p.i., immunohistochemical (IHC) analysis of the murine lungs further revealed that H3N2 and H5N1 viral antigens were most prominent in the bronchial and alveolar epithelium in ANP32B^+/+^ mice (Figure 2C). Reduction of H3N2 and H5N1 virus antigen in the lung of ANP32B^−/−^ mice was observed in both bronchial and alveolar epithelium particularly at 3 days p.i. (Figure 2C). These findings suggest that ANP32B deficiency delays viral replication especially during the early stages of infection, in line with the delayed weight loss shown in Figure 1.

The reduced capacity of H3N2 and H5N1 virus replication in ANP32B^−/−^ mice could be also verified in murine lung fibroblasts that were isolated from ANP32B^+/+^ and ANP32B^−/−^ mice (Figures 2D–F). Moreover, in murine cell culture, H5N1 virus replication was barely detectable in ANP32B^−/−^ cells compared to ANP32B^+/+^ cells (Figure 2E). Noteworthy, murine lung fibroblasts lacking ANP32A supported H3N2 virus replication comparably to ANP32A^+/+^ cells (Figures S2D,E). These findings strongly suggest that ANP32B, in contrast to ANP32A, acts as a positive factor of virus replication in murine cells.

### ANP32B Contributes to Viral Replication in Human Cells

**Figure 3:**
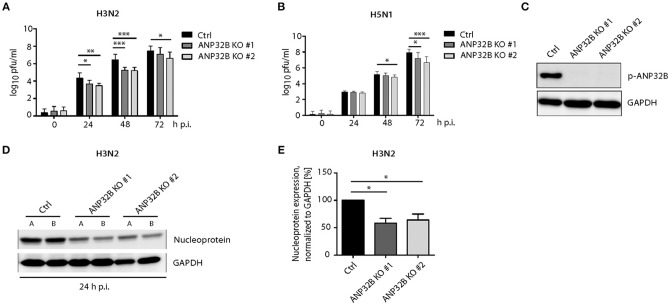
ANP32B contributes to influenza A virus replication in human cells. **(A,B)** Viral titers in H3N2 or H5N1 infected human HeLa cells deficient for ANP32B were determined by plaque test at the indicated time points. Two different knockout cell lines were used to exclude clonal artifacts (ANP32B KO #1 and ANP32B KO #2, respectively). **(C)** Knockout of ANP32B in two clonal HeLa cell lines was verified by Western blotting. **(D,E)** Expression of viral nucleoprotein (NP) in H3N2 infected HeLa cell lines deficient for ANP32B was analyzed by Western blotting at 24 h p.i. Quantification of viral NP expression from two independent experiments was performed using ImageJ software **(E)**. **(A–E)** Statistical significance was calculated using the two-tailed Student's *t-test* (*p ≤ 0.05, **p ≤ 0.01, ***p ≤ 0.001; ns, not significant).

In order to further characterize whether the ANP32B mode-of-action is restricted to murine cells or can be extrapolated to human cells as well, we assessed virus replication in human HeLa cells with a deleted ANP32B gene. In order to exclude clonal artifacts, we tested two individual ANP32B^−/−^ cell lines (ANP32B KO #1 and #2, respectively) as well as their control treated cells (Ctrl). Both, H3N2 as well as H5N1 virus replication was reduced in all ANP32B^−/−^ clones assessed, compared to Ctrl cells (Figures 3A–C). Furthermore, we analyzed the expression of viral nucleoprotein (NP) in H3N2 infected ANP32B knockout HeLa cells. Here, 24 h p.i., we observed significantly reduced NP levels compared to the Ctrl cells (Figures 3D,E). However, reduction of H3N2 and H5N1 replication in human cells was not as prominent as in murine cells (Figures 2D–F). These findings suggest that ANP32B acts as a positive factor of virus replication more prominently in murine cells.

### ANP32B Promotes Pro-inflammatory Cytokine Responses in the Murine Lung

**Figure 4:**
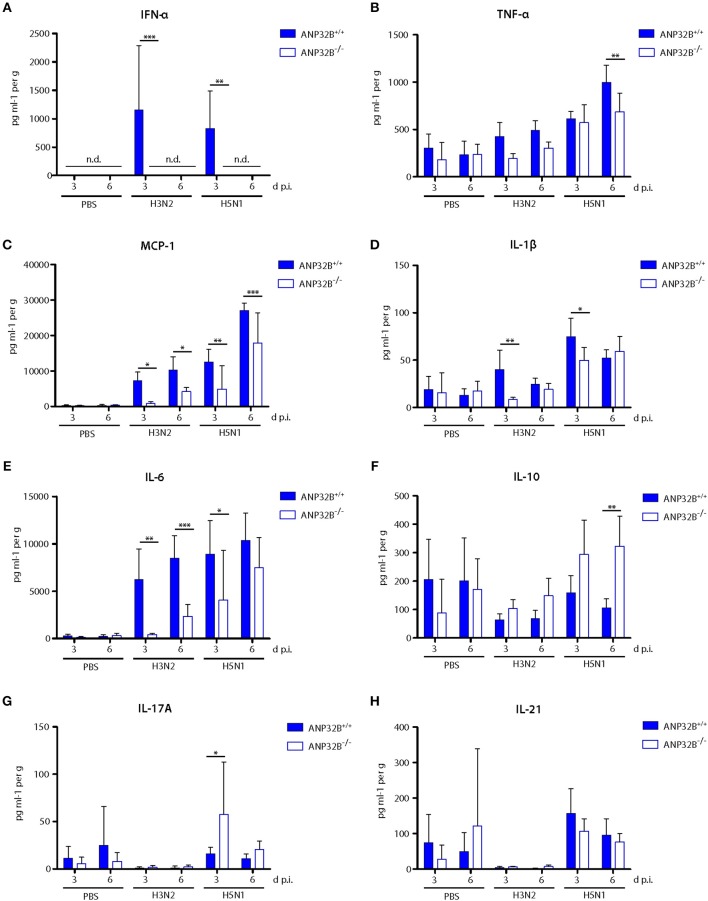
Altered cytokine and chemokine response in influenza A virus infected ANP32B^−/−^ mice. **(A–H)** ANP32B^+/+^ and ANP32B^−/−^ mice were either control treated with PBS or infected with 10^3^ pfu of a seasonal H3N2 subtype or a highly pathogenic H5N1 human isolate. At 3 and 6 d p.i., cytokine/chemokine expression levels were determined in lung homogenates using a multiplex immunoassay. Interferon-α (**A**, IFN-α), tumor necrosis factor α (**B**, TNF-α), monocyte chemotactic protein 1 (**C**, MCP-1), interleukin 1β (**D**, IL-1β), interleukin 6 (**E**, IL-6), interleukin 10 (**F**, IL-10), interleukin 17A (**G**, IL-17A), and interleukin 21 (**H**, IL-21). Presented are the concentrations measured for each cytokine/chemokine as mean ± SD for each group (*n* = 5–7). Statistical significance was calculated using two-way ANOVA with Bonferroni post-test (*p ≤ 0.05, **p ≤ 0.01, ***p ≤ 0.001; n.d., not detected).

We further investigated the induction of cytokine responses in the infected murine lungs as a key parameter of viral pathogenesis. In general, H3N2 and H5N1 infection induced IFN-α, TNF-α, MCP-1 (CCL-2), IL-1β, and IL-6 responses in ANP32B^+/+^ mice were reduced in ANP32B^−/−^ mice, particularly 3 days post infection (Figures 4A–E). Remarkably, IFN-α levels were below detection limits in ANP32B^−/−^ mice upon H3N2 and H5N1 infection compared to their ANP32B^+/+^ litter mates (Figure 4A). Conversely, IL-10 and IL-17A levels were significantly increased in H5N1 infected ANP32B^−/−^ mice at 6 or 3 d p.i., respectively, compared to ANP32B^+/+^ mice (Figures 4F,G). No differences in IL-10 and IL-17A responses could be detected in H3N2 infected ANP32B^+/+^ or ANP32B^−/−^ mice (Figures 4F,G). IL-21 induction was similar in H3N2 and H5N1 infected ANP32B^+/+^ or ANP32B^−/−^ mice (Figure 4H). As a control, we also assessed cytokine responses in ANP32A^+/+^ and ANP32A^−/−^ mice infected either with H3N2 or H5N1 influenza virus. No major differences could be observed among both groups (Figure S3). This further confirms that ANP32A is not essential for the induction of virus-induced cytokine responses. Our findings in ANP32B^+/+^ and ANP32B^−/−^ mice on the other hand suggest that ANP32B is required for the induction of a subset of pro-inflammatory cytokine responses (TNF-α, MCP-1, IL-1β, and IL-6), upon influenza infection in the murine lung. In line, an anti-inflammatory IL-10 response in the infected murine lungs was higher in mice lacking the ANP32B gene (Figure 4F). It is important to highlight that most prominently IFN-α induction was dependent on the presence of ANP32B suggesting its pivotal role in regulating antiviral immunity in the murine lung (Figure 4A).

### ANP32B Is Crucial for the Transcription of Inflammatory Genes in the Murine Lung

**Figure 5:**
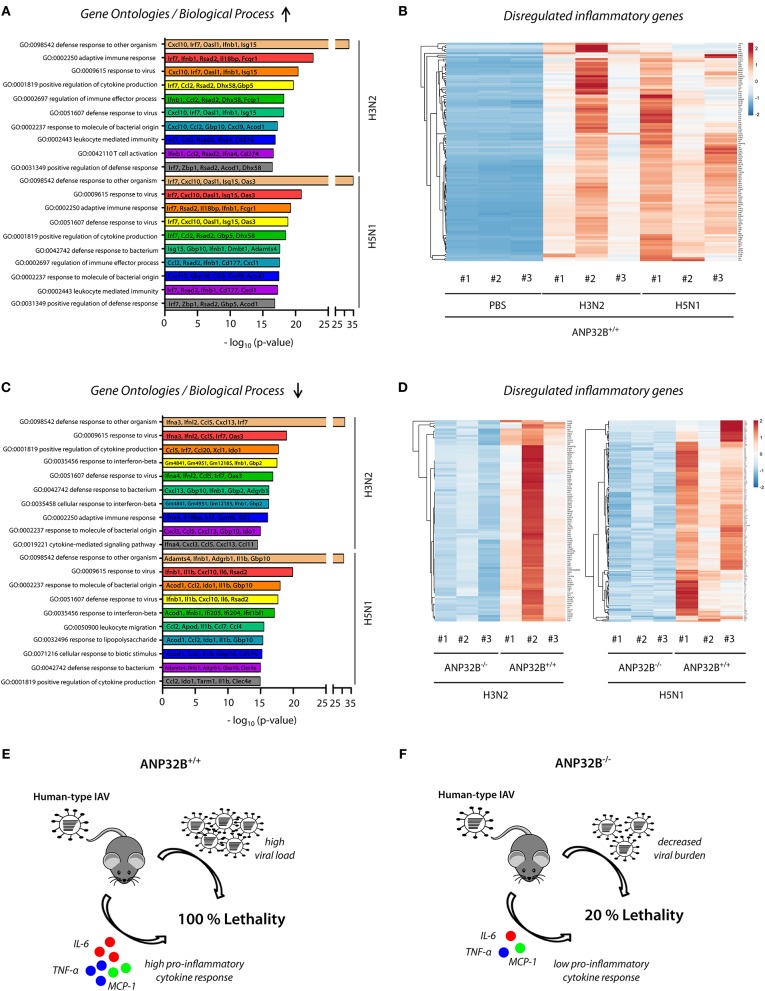
Global expression profile of inflammatory genes in ANP32B^−/−^ mice during influenza infection. ANP32B^+/+^ and ANP32B^−/−^ mice were either control treated with PBS or infected with 10^3^ pfu of a seasonal H3N2 subtype or a highly pathogenic H5N1 human isolate. At 3 d p.i., lungs were removed and total RNA was isolated and subjected to next generation sequencing (*n* = *3* animals per group). **(A)** Enriched top 10 gene ontologies (GOs) upon influenza A virus infection in ANP32B^+/+^ mice compared to PBS treated control mice. Top 5 of upregulated genes are shown for each respective GO. **(B)** Heatmap analysis of significantly upregulated genes during influenza A virus infection in ANP32B^+/+^ mice, based on GOs. Color code represents standard deviation from the mean of normalized expression values (*z-score*) within each group (*n* = 3 animals per group; cut-off: log2FoldChange ≥ 2). **(C)** Enriched top 10 gene ontologies (GOs) upon influenza A virus infection in ANP32B^+/+^ vs. ANP32B^−/−^ mice. Top 5 of downregulated genes are shown for each respective GO. **(D)** Heatmap analysis of significantly downregulated genes during influenza A virus infection in ANP32B^+/+^ vs. ANP32B^−/−^ mice, based on GOs. Color code represents standard deviation from the mean of normalized expression values within each group (*n* = 3 animals per group; cut-off: log2FoldChange ≤ −1). **(E,F)** Model: ANP32B^+/+^ mice succumb to influenza A virus infection due to high virus load and pro-inflammatory cytokine response (“cytokine storm”) **(E)**. In contrast, survival of ANP32B^−/−^ mice upon influenza A virus infection correlates with decreased viral burden and global suppression of pro-inflammatory cytokines responses **(F)**.

Significant changes in pro-inflammatory cytokine expression in mice lacking the ANP32B gene (Figure 4) prompted us to elucidate involved cellular pathways on a global level. Therefore, we determined the lung transcriptome of H3N2 or H5N1 IAV infected ANP32B^+/+^ and ANP32B^−/−^ mice at 3 days post infection. First, we analyzed the differential gene expression in virus infected ANP32B^+/+^ mice compared to the PBS treated ANP32B^+/+^ control mice (Figures 5A,B; Figures S4A,B). As expected, pro-inflammatory genes (e.g., *Ifnb1, MCP-1/Ccl2, Cxcl10)* associated with a response to viral infection were most prominently upregulated in H3N2 and H5N1 infected mice, as evident in gene ontology (GO) analyses (Figure 5A; Tables S1, S2). *Mx1, Irf7* and other established target genes of the antiviral transcription factor NF-κB were significantly upregulated in infected mice. Only a few genes were found to be downregulated upon infection (Figures S4A,B; Tables S1, S2). Heat map analyses of genes presented in the top 10 of altered GOs (Figure 5A) clearly differentiate the virus infected mice from the PBS treated control mice (Figure 5B). These data highlight the important contribution of high pro-inflammatory cytokine responses, also known as “cytokine storm,” to lethal influenza outcome in mice.

Next, we assessed the transcriptomic pattern in infected ANP32B^−/−^ vs. ANP32B^+/+^ mice (Figures 5C,D). MA plot analyses reveal a shift towards a downregulated gene expression profile in ANP32B^−/−^ mice during virus infection, compared to ANP32B^+/+^ mice (Figures S4C,D). Interestingly, according to GO terms, a majority of these downregulated genes is associated with the host pro-inflammatory response to viral infection (e.g., *Ccl2, IL-6, IL-1*β) (Figure 5C). In H5N1 infected ANP32B^−/−^ mice, a higher number of significantly downregulated genes was identified, compared to H3N2 infected mice (521 vs. 320 genes, respectively). Furthermore, while certain genes were repressed during both virus infections (e.g., *Irf7* or *Ifnb1*), expression of others was more prominently reduced upon H5N1 infection (e.g., *IL-1*β, *IL-6, Ccl2*) (Figure 5C; Tables S3, S4). These results suggest that common as well as different ANP32B-regulated pathways contribute to disease outcome in a virus subtype-specific manner. Of note, H3N2 infection in ANP32B^−/−^ mice did not result in any significantly up-regulated genes, while H5N1 infection was associated with an increase in expression of genes regulating cilia movement and assembly (Tables S3, S4). Overall, a distinct transcriptional profile characterized by a global reduction of antiviral gene expression was identified that distinguishes the ANP32B^−/−^ mice from their ANP32B^+/+^ litter mates during influenza infection (Figure 5D). Noteworthy, we observed some variability between the three different H3N2 IAV infected ANP32B^+/+^ mice regarding their individual transcriptional profiles (Figure 5D, left panel). However, this seeming discrepancy changed when the transcriptional alterations in mouse #2 were normalized against its higher than average replication kinetics and cytokine levels (Figure S5). Thus, the transcriptomic profile shown in Figure 5D (left panel) reflects biological variability and needs to be regarded in the context of viral infection efficiency.

In conclusion, the transcriptional landscape of the IAV infected murine lung strongly suggests that ANP32B plays a crucial role in the regulation of pro-inflammatory gene expression that ultimately dictates influenza disease outcome in mice (Figures 5E,F).

## Discussion

Elucidating virus-host interactions is the basis for understanding viral pathogenesis, which in turn is crucial for the development of antiviral treatment strategies. Zoonotic viruses utilize host factors very specifically to facilitate animal-to-man transmission. Influenza A virus avian-mammalian transmission is mediated via complex and dynamic interactions of viral and cellular proteins (25). Recently, it was postulated that the ANP32A and ANP32B proteins play a crucial role in IAV interspecies transmission (10). However, these factors were initially identified and studied in cell culture only.

In this study, we provide first evidence regarding the *in vivo* relevance of these factors by using mice that lack either the ANP32A or ANP32B gene. Here, we show that ANP32A is not required for high-titer IAV replication, virus-induced cytokine responses or pathogenicity in mice. At first sight, these observations seem to contradict the initial studies performed in human cell lines. There, using specific siRNAs, ANP32A and ANP32B were required for high human-type (PB2 627K) polymerase activity (10). However, more recently, these findings were challenged by two additional studies reporting that PB2 627K mammalian-adapted IAV replicate efficiently in human ANP32A knockout cells (13, 26). In the study by Staller et al. the authors further show that an aspartate at position 130 is associated with the species dependent ability of ANP32 proteins to promote mammalian-type IAV polymerase activity and replication in human cells (13). Murine ANP32A contains an alanine at this position, which might explain why it has lost its ability to support IAV replicative fitness and pathogenicity in mice. Therefore, species-specific differences in ANP32A seem to hamper the use of small animal models to study IAV pathogenesis, in contrast to other previously reported host factors that act by functionally conserved mechanisms in mice and in human cells (3, 7). Thus, future studies will be required to assess a potential role of ANP32A on influenza disease outcome in humans.

In contrast, human and murine ANP32B seem to exert a potent role in promoting PB2 627K IAV replication in human cell lines and in mice, respectively (13, 27). However, ANP32B was less potent in promoting IAV replication in human cell lines compared to mice. Thus, future studies assessing the role of ANP32B in human primary cells would be required. In mice on the other hand, ANP32B is essential for high virus pathogenicity. To-date, the mechanism underlying the ANP32B mode-of function is still unclear. Here, we provide first evidence that ANP32B is crucial for the transcriptomic regulation of a series of pro-inflammatory cytokines with partially antiviral functions in the murine lung. Elevated cytokine response in the murine lung upon human H3N2 and avian H5N1 infection was highly dependent on the presence of ANP32B. Thus, mice lacking ANP32B lost their ability to support high-titer virus replication, antiviral gene expression in the lung and high pathogenicity in mice. These findings may support the concept that ANP32B possesses an immune-regulatory function. Indeed, in a recent study by Chemnitz et al. ANP32B was shown to shape the adaptive immune response in an experimental autoimmune encephalomyelitis model, albeit the underlying molecular mechanisms are still unclear (18). Based on proposed cellular functions of ANP32B, we speculate that it could either directly interfere with intracellular signaling cascades leading to innate immune activation, or actively remodel the chromatin landscape through its role as a histone chaperone (28, 29). Others have shown that ANP32B acts as an adaptor for HuR/Crm1 mediated nuclear export of CD83 mRNA, an established maker for activated dendritic cells, which could indirectly modulate the link between innate and adaptive immunity (20). However, future studies are required to dissect the direct impact of ANP32B on virus replication and/or innate immune responses.

In summary, this study forms an important basis for our understanding of molecular pathways underlying IAV interspecies transmission and pathogenicity in mammals. Furthermore, the data obtained herein might contribute to the development of novel treatment strategies against severe influenza.

## Data Availability Statement

The datasets generated for this study can be found in the European Nucleotide Archive (ENA) via the accession number PRJEB35060.

## Ethics Statement

The animal study was reviewed and approved by Behörde für Stadtentwicklung und Umwelt Hamburg, licensing number: 08/17.

## Author Contributions

SB, SS-B, and GG designed the experiments and analyzed the data. SB conducted all animal experiments and was involved in all subsequent analyses. MZ contributed to viral replication kinetics in human cells. VP performed imaging of histological slides. TG and AG carried out secondary analyses of raw NGS data. PR and TM generated and provided the ANP32A knockout mice. SB and GG wrote the manuscript.

### Conflict of Interest

The authors declare that the research was conducted in the absence of any commercial or financial relationships that could be construed as a potential conflict of interest.
