# Supplementary material for: ANP32B Deficiency Protects Mice From Lethal Influenza A Virus Challenge by Dampening the Host Immune Response
Source: Front Immunol. 2020 Mar 13;11:450. doi: 10.3389/fimmu.2020.00450 (PMC7083139; doi:10.3389/fimmu.2020.00450)
Supplement: Supplementary file 1 [file Data_Sheet_1.PDF]

Supplemental Table Legends

Table S1. Differentially regulated genes in ANP32B$^{+/+}$ mice upon H3N2 influenza A virus infection vs. PBS control. ANP32B$^{+/+}$ mice were either control treated with PBS or infected with 10^3 pfu of a seasonal H3N2 subtype. At 3 d p.i., lungs were removed and total RNA was isolated and subjected to next generation sequencing. Results were further evaluated manually as described in *Materials and Methods*. Shown are significantly up- or downregulated genes (log2FoldChange ≥ 1 or ≤ -1, respectively), or unfiltered results, in H3N2 infected mice vs. PBS-treated control mice. Genes were sorted alphabetically and parameters shown include *GeneSymbol, EntrezID, log2FoldChange, p-value and FDR.*

Table S2. Differentially regulated genes in ANP32B$^{+/+}$ mice upon H5N1 influenza A virus infection vs. PBS control. ANP32B$^{+/+}$ mice were either control treated with PBS or infected with 10^3 pfu of a highly pathogenic H5N1 human isolate. At 3 d p.i., lungs were removed and total RNA was isolated and subjected to next generation sequencing. Results were further evaluated manually as described in *Materials and Methods*. Shown are significantly up- or downregulated genes (log2FoldChange ≥ 1 or ≤ -1, respectively), or unfiltered results, in H5N1 infected mice vs. PBS-treated control mice. Genes were sorted alphabetically and parameters shown include *GeneSymbol, EntrezID, log2FoldChange, p-value and FDR.*

Table S3. Differentially regulated genes in ANP32B$^{+/+}$ vs. ANP32B$^{-/-}$ mice upon H3N2 influenza A virus infection. ANP32B$^{+/+}$ or ANP32B$^{-/-}$ mice were either control treated with PBS or infected with 10^3 pfu of a seasonal H3N2 subtype. At 3 d p.i., lungs were removed and total RNA was isolated and subjected to next generation sequencing. Results were further evaluated manually as described in *Materials and Methods*. Shown are significantly up- or downregulated genes (log2FoldChange ≥ 1 or ≤ -1, respectively), or unfiltered results, in H3N2 infected ANP32B$^{+/+}$ vs. ANP32B$^{-/-}$ mice. Genes were sorted alphabetically and parameters shown include *GeneSymbol, EntrezID, log2FoldChange, p-value and FDR.*

Table S4. Differentially regulated genes in ANP32B$^{+/+}$ vs. ANP32B$^{-/-}$ mice upon H5N1 influenza A virus infection. ANP32B$^{+/+}$ or ANP32B$^{-/-}$ mice were either control treated with PBS or infected with 10^3 pfu of a highly pathogenic H5N1 human isolate. At 3 d p.i., lungs were removed and total RNA was isolated and subjected to next generation sequencing. Results were further evaluated manually as described in *Materials and Methods*. Shown are significantly up- or downregulated genes (log2FoldChange ≥ 1 or ≤ -1, respectively), or unfiltered results, in H5N1 infected ANP32B$^{+/+}$ vs. ANP32B$^{-/-}$ mice. Genes were sorted alphabetically and parameters shown include *GeneSymbol, EntrezID, log2FoldChange, p-value and FDR.*
