# Supplementary material for: ANP32B Deficiency Protects Mice From Lethal Influenza A Virus Challenge by Dampening the Host Immune Response
Source: Front Immunol. 2020 Mar 13;11:450. doi: 10.3389/fimmu.2020.00450 (PMC7083139; doi:10.3389/fimmu.2020.00450)
Supplement: Supplementary file 2 [file Data_Sheet_2.PDF]

Figure S1

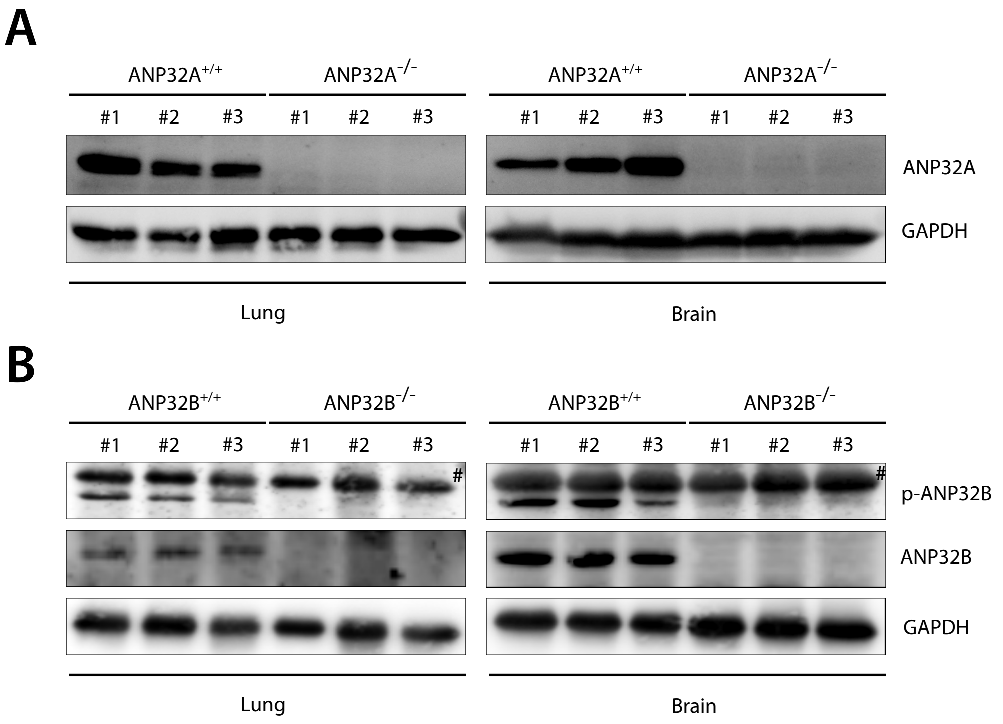
A
ANP32A+/+
ANP32A-/-
#1
#2
#3
#1
#2
#3
ANP32A
GAPDH
Lung
Brain
B
ANP32B+/+
ANP32B-/-
#
p-ANP32B
ANP32B
GAPDH
Lung
Brain

Figure S1. Confirmation of ANP32 protein knockout in the lung and brain of ANP32A$^{-/-}$ and ANP32B$^{-/-}$ mice (accompanying Figure 1). Lung and brain homogenates were prepared from ANP32A$^{-/-}$ (**A**) or ANP32B$^{-/-}$ (**B**) mice as well as their corresponding wild type litter mates (ANP32A$^{+/+}$ and ANP32B$^{+/+}$, respectively). ANP32 protein expression was analyzed by Western blotting using protein-specific antibodies. n = 3 animals per genotype. #, unspecific band.

Figure S2

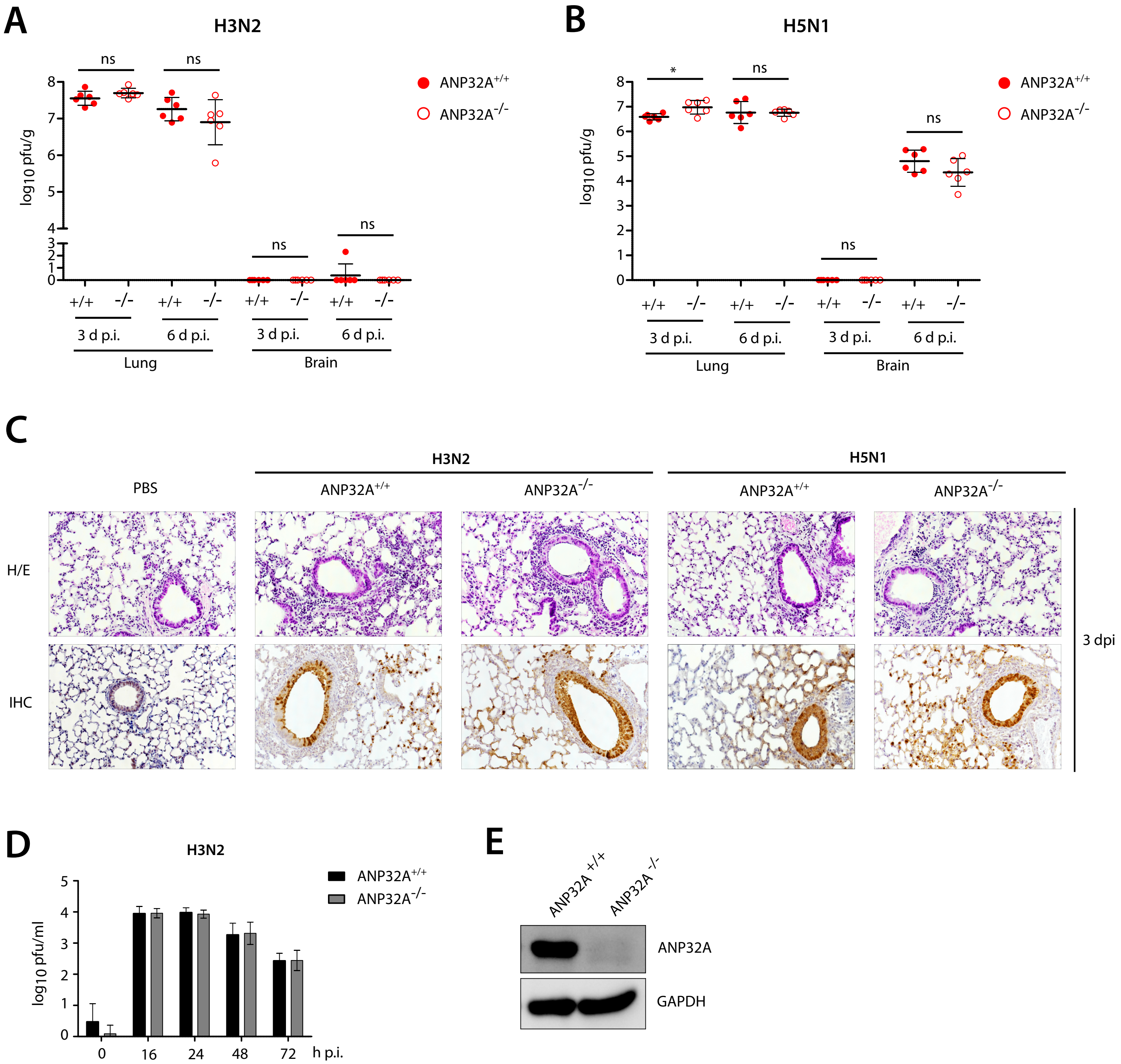

A
H3N2
ns
ns
ns
ns
log10 pfu/g
ANP32A+/+
ANP32A-/-
+/+
-/-
3 d p.i.
6 d p.i.
Lung
Brain
B
H5N1
*
ns
ns
ns
C
PBS
H3N2
H5N1
ANP32A+/+
ANP32A-/-
H/E
IHC
3 dpi
D
H3N2
log10 pfu/ml
h p.i.
E
ANP32A +/+
ANP32A -/-
ANP32A
GAPDH

Figure S2. Murine ANP32A knockout does not affect influenza A virus replication in mice (accompanying Figure 2). (**A-C**) ANP32A$^{+/+}$ and ANP32A$^{-/-}$ mice were either control treated with PBS or infected with 10^3 pfu of a seasonal H3N2 subtype (**A**) or a highly pathogenic H5N1 human isolate (**B**). (**A-B**) Viral titers were determined 3 and 6 days p.i. in lung and brain of infected animals. No virus was detected in PBS infected mice (*n* = 5). Presented are individual organ titers for each animal as well as the means ± SD for each group (*n* = 5-6). (**C**) At 3 d p.i., lungs from infected animals were removed and immunohistochemically (IHC) stained for viral NP antigen. Additionally, hematoxylin and eosin (H/E) staining was performed. Shown are representative images for each group (*n* = 5). Original magnification, 10x. (**D**) Viral titers in H3N2 infected murine lung fibroblasts deficient for ANP32A were determined by plaque test at the indicated time points (h p.i., hours post infection). (**E**) Knockout of ANP32A in murine lung fibroblasts was confirmed by Western blotting.

Figure S3

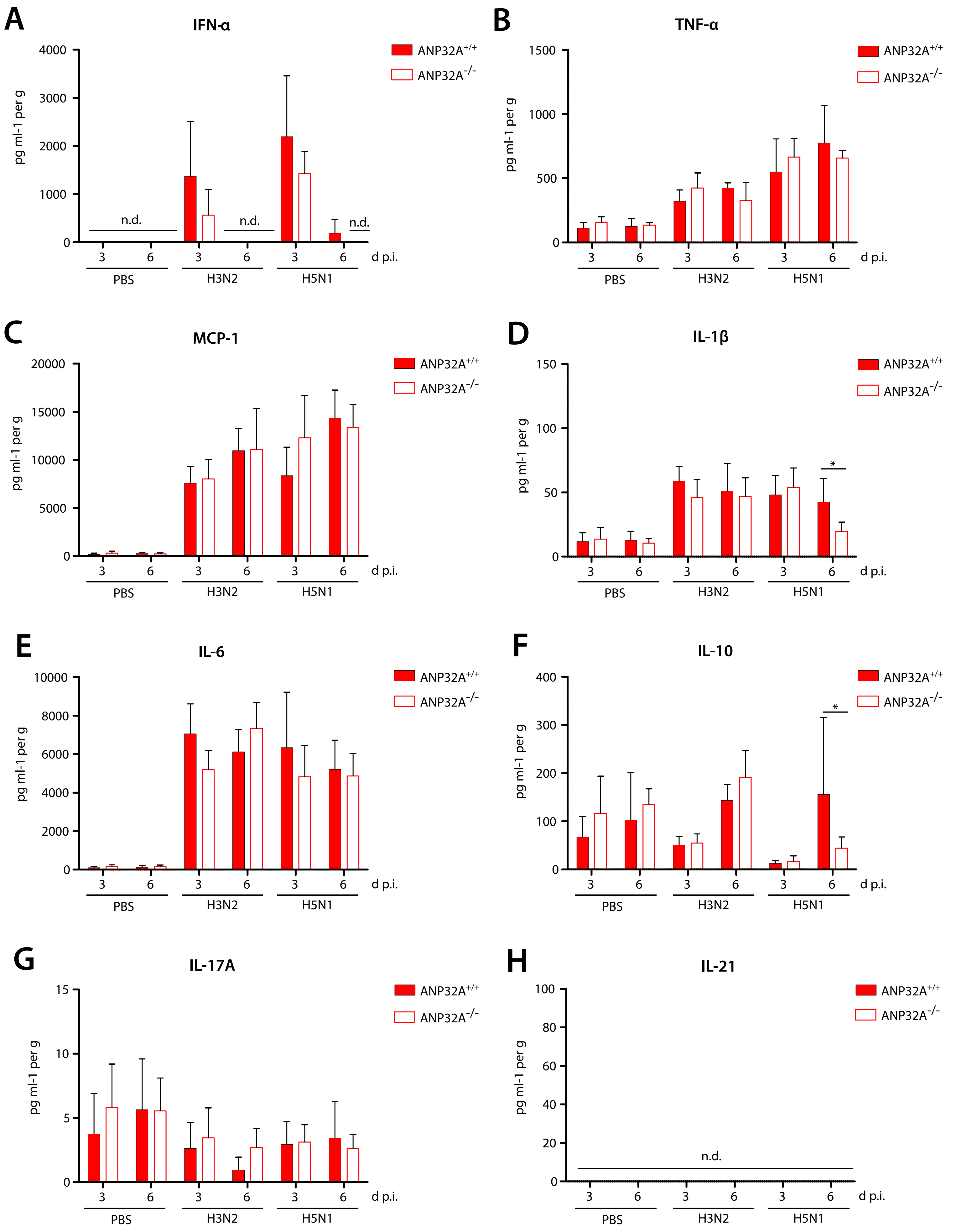

A
IFN-α
B
TNF-α
C
MCP-1
D
IL-1β
E
IL-6
F
IL-10
G
IL-17A
H
IL-21
ANP32A+/+
ANP32A-/-
pg ml-1 per g
n.d.
*
3
6
d p.i.
PBS
H3N2
H5N1

Figure S3. Comparable cytokine and chemokine response in influenza A virus infected ANP32A$^{+/+}$ and ANP32A$^{-/-}$ mice (accompanying Figure 4). (**A-H**) ANP32A$^{+/+}$ and ANP32A$^{-/-}$ mice were either control treated with PBS or infected with 10^3 pfu of a seasonal H3N2 subtype or a highly pathogenic H5N1 human isolate. At 3 and 6 d p.i., cytokine/chemokine expression levels were determined in lung homogenates using a multiplex immunoassay. Interferon-α (**A**, IFN-α), tumor necrosis factor α (**B**, TNF-α), monocyte chemotactic protein 1 (**C**, MCP-1), interleukin 1β (**D**, IL-1β), interleukin 6 (**E**, IL-6), interleukin 10 (**F**, IL-10), interleukin 17A (**G**, IL-17A), and interleukin 21 (**H**, IL-21). Presented are the concentrations measured for each cytokine/chemokine as mean ± SD for each group (n = 5-7). Statistical significance was calculated using two-way ANOVA with Bonferroni post-test (* $p \leq 0.05$, ** $p \leq 0.01$, *** $p \leq 0.001$; n.d., not detected).

Figure S4

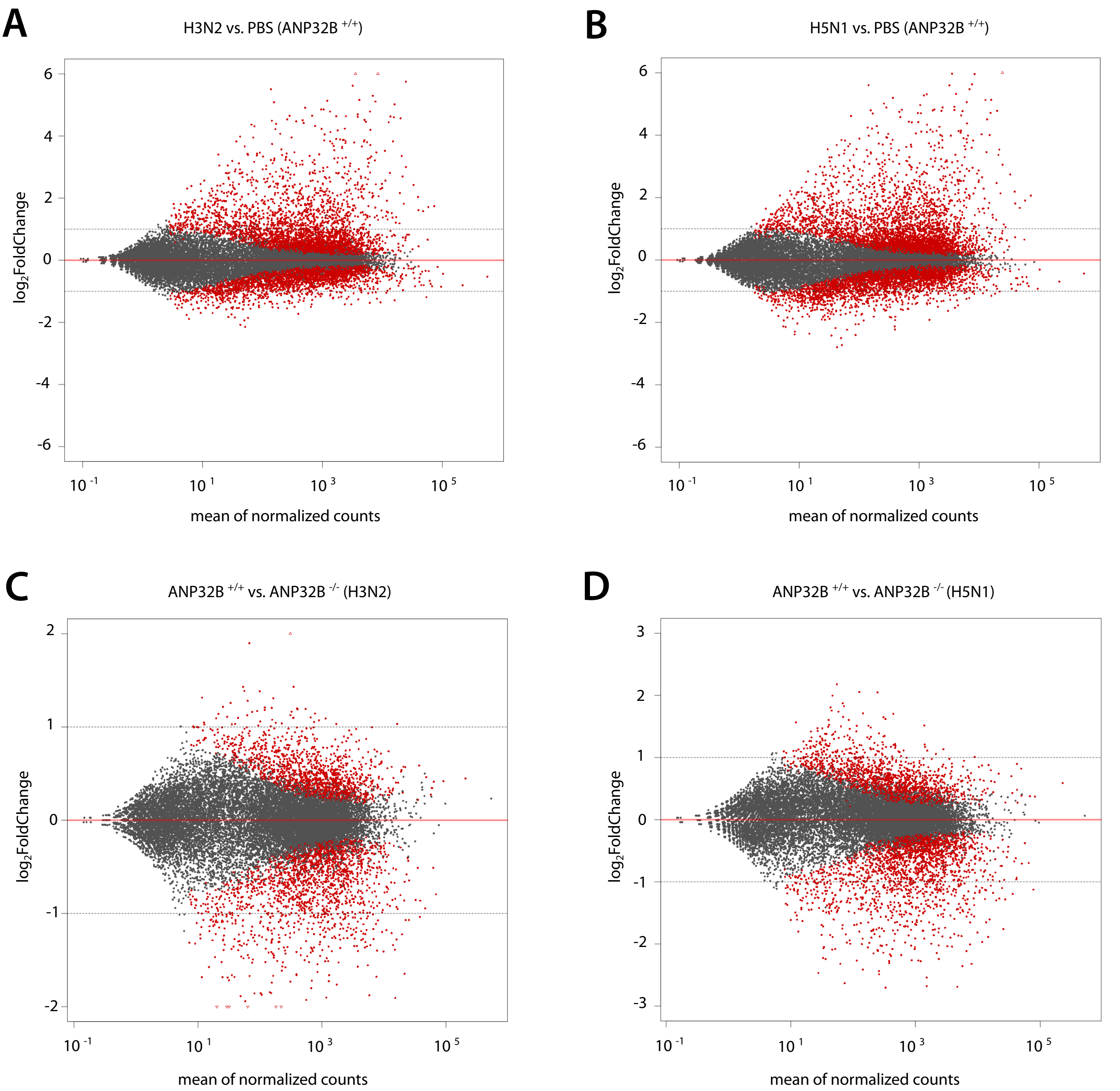
A
H3N2 vs. PBS (ANP32B +/+)
log2FoldChange
mean of normalized counts
B
H5N1 vs. PBS (ANP32B +/+)
log2FoldChange
mean of normalized counts
C
ANP32B +/+ vs. ANP32B -/- (H3N2)
log2FoldChange
mean of normalized counts
D
ANP32B +/+ vs. ANP32B -/- (H5N1)
log2FoldChange
mean of normalized counts

Figure S4. Differentially regulated genes in influenza A virus infected ANP32B$^{+/+}$ and ANP32B$^{-/-}$ mice (accompanying Figure 5). ANP32B$^{+/+}$ and ANP32B$^{-/-}$ mice were either control treated with PBS or infected with 10^3 pfu of a seasonal H3N2 subtype or a highly pathogenic H5N1 human isolate. At 3 d p.i., lungs were removed and total RNA was isolated and subjected to next generation sequencing. (**A-D**)**,** MA plot analysis showing all differentially regulated genes in infected ANP32B$^{+/+}$ and ANP32B$^{-/-}$ mice 3 days post infection, plotted as log2FoldChange over the mean of normalized counts for each experimental group (n = 3 animals per group). Cut-off (log2FoldChange ≥ 1 or ≤ -1) for significantly disregulated genes is indicated with dashed lines. (**A**) H3N2 infection vs. PBS control in ANP32B$^{+/+}$ mice; (**B**) H5N1 infection vs. PBS control in ANP32B$^{+/+}$ mice; (**C**) H3N2 infection in ANP32B$^{+/+}$ vs. ANP32B$^{-/-}$ mice; (**D**) H5N1 infection in ANP32B$^{+/+}$ vs. ANP32B$^{-/-}$ mice.

Figure S5

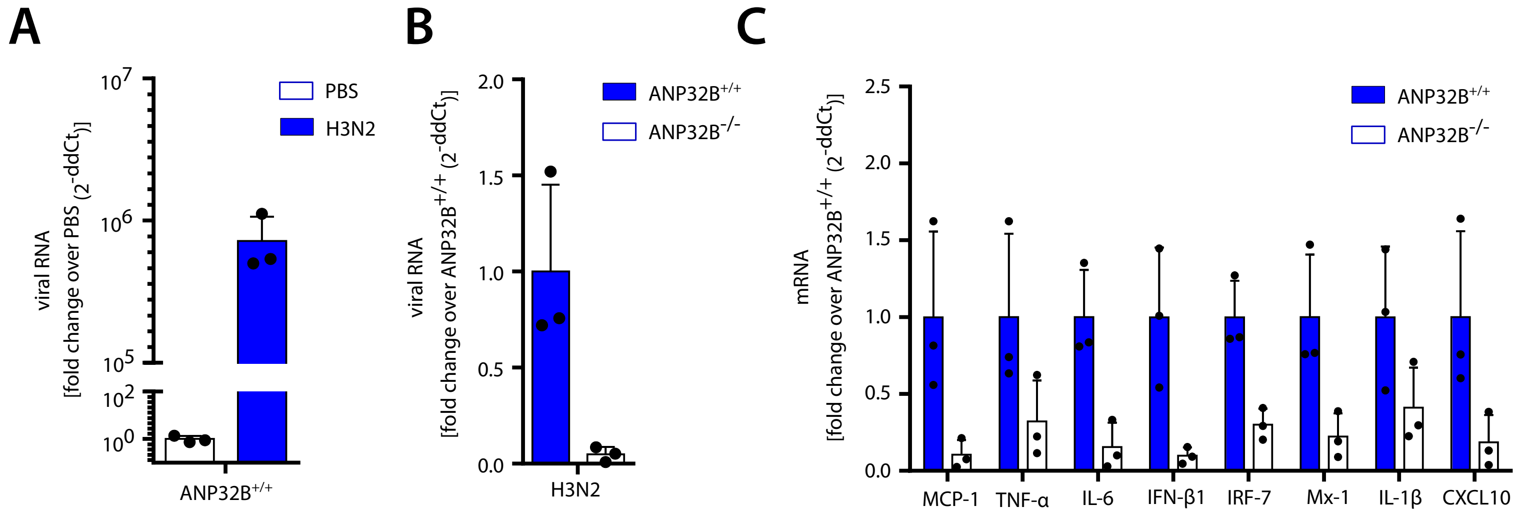

A
B
C
PBS
H3N2
ANP32B+/+
ANP32B-/-
viral RNA
[fold change over PBS (2-ddCt)]
[fold change over ANP32B+/+ (2-ddCt)]
mRNA
H3N2
MCP-1
TNF-α
IL-6
IFN-β1
IRF-7
Mx-1
IL-1β
CXCL10

Figure S5. Viral replication and cytokine response in infected ANP32B$^{+/+}$ and ANP32B$^{-/-}$ mice (accompanying Figure 5). ANP32B$^{+/+}$ and ANP32B$^{-/-}$ mice were either control treated with PBS or infected with 10^3 pfu of a seasonal H3N2 subtype. At 3 d p.i., lungs were removed and total RNA was isolated. **(A)** Expression of viral nucleoprotein (NP) RNA in infected ANP32B$^{+/+}$ mice, compared to PBS treated control mice. **(B)** Expression of viral NP RNA in infected ANP32B$^{+/+}$ vs. ANP32B$^{-/-}$ mice. **(C)** mRNA expression of a series of cytokines/chemokines (MCP-1, TNF-α, IL-6, IFN-β1, CXCL10, IL-1β), antiviral transcription factors (IRF-7) and effector proteins (Mx1) in infected ANP32B$^{+/+}$ vs. ANP32B$^{-/-}$ mice.
